# Prevalence and risk factors of low back pain among school age children in Iran

**DOI:** 10.15171/hpp.2017.39

**Published:** 2017-09-26

**Authors:** Iman Dianat, Arezou Alipour, Mohammad Asghari Jafarabadi

**Affiliations:** ^1^ Department of Occupational Health and Ergonomics, Tabriz University of Medical Sciences, Tabriz, Iran; ^2^Road Traffic Injury Research Center, Tabriz University of Medical Sciences, Tabriz, Iran; ^3^Department of Statistics and Epidemiology, Faculty of Health, Tabriz University of Medical Sciences, Tabriz, Iran

**Keywords:** Musculoskeletal, Children, Psychological, Classroom furniture, Schoolbag

## Abstract

**Background:** Most available data on the prevalence and characteristics of back pain in schoolchildren is related to industrialised and developed countries. The aim of this study was to investigate the prevalence of low back pain (LBP) and potential risk factors among schoolchildren and adolescents in a developing country, Iran.

**Methods:** A cross-sectional study was conducted among 1611 Iranian schoolchildren aged 11–14 years. A self-complete questionnaire was used to assess LBP prevalence, physical leisure activities, school-related and psychosocial factors.

**Results:** The prevalence of LBP was 34.3%. Female gender (odds ratio [OR] = 1.57, 95% CI:1.28–1.94), family member with back pain (OR = 1.82, 95% CI: 1.40–2.36), difficulty in viewing the (black)board (OR = 1.50, 95% CI: 1.13–1.99), too much homework (OR = 1.47, 95% CI:1.09–1.99), time spend carrying a schoolbag (min/d) (OR = 1.37, 95% CI: 1.01–1.85), and psychosocial factors (emotional symptoms) (OR = 2.28, 95% CI: 1.54–3.39) were independently associated with LBP. Physical activity, having a job, watching television, using a computer, playing games and schoolbag weight had no effect.

**Conclusion:** It can be concluded that both physical and psychosocial factors influenced the risk for LBP, but emotional symptoms had a stronger association with LBP than physical factors. Knowledge about LBP in school children and adolescents could be important in assessment and treatment of such symptoms in this population.

## Introduction

Recent evidence has demonstrated that low back pain (LBP) is frequent among schoolchildren and youth.^1-4^ The reported incidence of back pain in schoolchildren and youth ranges from 7% to 74% in the literature.^5,6^ Evidence suggests that back pain in childhood and adolescence is a contributory factor for the increased incidence of such complaints in adulthood.^7,8^ Thus, there appears to be a need to improve the understanding of the risk factors associated with back pain among children and adolescents.

A number of factors may contribute to the development of musculoskeletal complaints in schoolchildren. The risk and protective factors of back pain in children and adolescents can be divided into individual, physical and psychosocial factors.^2-4,9^ Design of classroom furniture seems to be one of the contributing factors to the development of musculoskeletal pain among schoolchildren.^4,9,10^ Though not conclusive, there is some evidence that loads carried by schoolchildren (schoolbag or backpack carriage) may be a factor contributing to musculoskeletal complaints among this age group.^6,11-13^ There are also contradictory findings in the literature regarding the association between musculoskeletal symptoms and physical leisure activity and life style factors (i.e. playing sport, watching TV, using a computer, etc.) in schoolchildren with some studies showing a positive association,^9^ while other show no association.^4^ Some studies have also noted a significant association between musculoskeletal symptoms and psychosocial factors.^2-4,9,14^

Most available data on the prevalence and characteristics of back pain in schoolchildren and adolescents is related to industrialised and developed countries,^5^ while there is limited information in this area in developing and low-income countries. The results of the studies conducted in the developed countries may not necessarily be generalizable to other countries or settings, where the cultural backgrounds or educational systems may be different. Furthermore, most previous studies have not considered the simultaneous effects of various factors (i.e. school-related factors such as classroom furniture and layout design, etc.) on the occurrence of back pain in schoolchildren.^12,14-16^ As a consequence, the effects of risk factors are not consistent and conclusive.

This present study was conducted to evaluate the occurrence of LBP and its contributing risk factors (i.e. demographic and physical leisure activity, school-related, and psychosocial factors) among schoolchildren in a developing country, Iran. The intention was to consider a broader combination of factors (in multivariate context) that influence LBP in school-aged children and also widen the discussion to more countries.

## Material and Methods

### Study population and procedure

This cross-sectional analytical study was performed in the city of Tabriz, with a population of about 1.58 million. Tabriz is the fourth major city in Iran and the capital of East Azerbaijan province in North-western Iran. The study was conducted between October 2014 and March 2015. To compute sample size, the primary information for the prevalence of LBP in school children was estimated through a pilot study, which involved a sample of 100 students randomly selected form the study population. Considering the minimum value of odds ratio (OR) of 1.2, 95% confidence level and 80% power, the minimum required sample size was estimated as 825 using G-Power software (version 3.1.2). The sample size was finally increased to 1650 in view of the design effect of 2.0 for cluster sampling. Those children aged 11–14 years and were in grades 6–8 were included in the study. A 3-stage sampling method was adopted to obtain a representative sample of schoolchildren. In the first stage, 5 educational districts were selected as strata. In the second stage, a total of 40 schools were chosen randomly from these districts, which included 4 girl’s schools and 4 boy’s schools from each district. Lastly, the study sample was chosen randomly (using a simple random sampling scheme) from each school. Before the children’s participation in the study, permission to approach the selected schools was taken from the Education Department of Tabriz as well as the school authorities involved. The schoolchildren were also given letters requesting parental permission for them to participate in the study.

### Data collection

The data collection was performed using a comprehensive questionnaire (as described below) as well as measurement of height (using a portable stadiometer) and weights (including body weight and schoolbag weight measurements using a digital electronic scale). The questionnaire and measuring process took approximately 20 minutes to complete in total. All of the data were collected by one of the authors (A.A) interviewing the participating students in order to avoid inter-observer variation and non-response/non-participation bias.

A questionnaire, consisted of 4 sections, was developed by the authors. The demographic and physical leisure activity variables (including gender, age, educational grade in school, weight, height, body mass index (BMI as kg/m^2^), having a family member with LBP as well as playing sport, using a computer, playing games, watching TV and having a part time job) were recorded in the first part of the questionnaire. In the second part of the questionnaire, LBP was evaluated by using a pre-shaded manikin picture showing the low back area and the following question^14^: “During the past month have you experienced any pain or ache in the low back area which has lasted for one day or longer?” (response alternatives: yes/no). The severity of complaints (using a scale of 0–5, where 0 = no pain and 5 = very high pain), as well as disruption of normal activities, absent from school and visiting a doctor due to LBP were also recorded.

The next part of the questionnaire contained school-related items and provided information about the suitability and comfort of the classroom furniture (based on a modification of the Chair Feature Checklist,^17^ as well as classroom layout design, homework and schoolbag carriage variables, which were based on the literature^4,9,13^ and prior knowledge of physical factors that might influence back pain in children and adolescents. For the classroom furniture and layout design items, the “just right” or “very easy” response alternative was considered as being appropriate.

Finally, in the last part of the questionnaire, psychosocial factors were evaluated by the Strengths and Difficulties Questionnaire (SDQ).^18^ This tool has been used previously among schoolchildren and its validity and reliability have been confirmed.^2-4,9^ The English version of the SDQ has already been translated into Farsi (Iranian language), tested and revised by Ghanizadeh et al.^19^ The SDQ is a behavioural screening questionnaire that evaluates 5 dimensions including hyperactivity (5 items), emotional symptoms (5 items), peer problems (5 items) and conduct problems (5 items) (which are related to negative behaviour or “difficulties”) and prosocial behaviour (5 items) (which shows positive behaviour or “strengths”). Each item is scored on a scale ranging from 1 “Not True” to 3 “Certainly True” and the score for each subscale ranges from 3 to 15. A higher score on the “strengths” subscale (i.e. prosocial behaviour) indicates positive behaviour, while higher scores on the 4 “difficulties” subscales indicate negative behaviour.

The questionnaire used in this study was evaluated through a pilot study on a sample of 170 school children. Some minor, non-substantive, revisions related to wording and clarity was then made to some of the questionnaire items according to their feedback. Moreover, the test-retest stability (with a 2-week interval between sessions) of the questionnaire used in the present study showed an acceptable degree of reliability (Kappa coefficients ranged from 0.72 to 0.96).

### Statistical analysis

Statistical analysis of the data was performed using SPSS version 14 software (SPSS Inc., Chicago, IL, USA). The relationship between the prevalence of LBP and study variables was initially evaluated using univariate logistic regression analyses. All variables significant at *P* < 0.10 in the univariate logistic regression analysis were subsequently included in one multivariate analysis using a backward stepwise logistic regression model. The OR and 95% CI was also calculated from logistic regression analyses. The model assumptions including collinearity (i.e. wide confidence intervals) and the presence of outliers were checked and were not in violation. The fit of the logistic regression model was confirmed by the Hosmer-Lemeshow goodness-of-fit test. *P* values <0.05 were considered statistically significant.

## Results

### Description of the study sample

A total of 1700 schoolchildren were approached, of which 1627 (response rate = 95.7%) accepted to respond to the questionnaire. Of those that responded, 16 were excluded for incomplete questionnaires. Therefore, the responses from 1611 (860 girls and 751 boys) were included in the analyses.

### Demographic and other details 

The participants’ characteristics were as follows (mean and standard deviation [SD]): age, 13.4 (0.89) years; weight, 49.7 (11.36) kg (boys: 50.2 [12.28] kg; girls: 49.3 [10.46] kg); height, 158.0 (10.76) cm (boys: 158.1 [12.57] cm; girls: 157.8 [8.86) cm]; BMI, 19.9 (3.83) kg/m^2^ (boys: 20.1 [4.11] kg/m^2^; girls: 19.7 [3.55] kg/m^2^). Characteristics of the physical leisure activity, use of schoolbags and SDQ scores among the schoolchildren are presented in Table 1.

### Characteristics of the LBP

The 1-month period prevalence of LBP was 34.3% (n = 553). The severity ratings for the low back area were found to differ significantly between boys (mean = 2.5, SD = 1.25) and girls (mean = 3.1, SD = 1.32) (*P *< 0.01). Other characteristics of the LBP among the study population are shown in Table 2.

### Univariate analyses

Table 3 shows the associations between individual and physical leisure activity factors and the occurrence of LBP in the univariate analyses. LBP was reported by girls (39%) significantly more frequently than by boys (29%) (OR = 1.57, 95% CI: 1.28–1.94, *P *< 0.001). Family member with back pain was also associated with LBP (OR = 1.83, 95% CI: 1.48–2.27, *P *< 0.001). Other factors including age, BMI and having a job, as well as time spent on sport activities, watching television, using a computer or playing games were not associated with LBP.

The association between school-related variables and LBP in the univariate analysis is shown in Table 4. The following school-related variables showed a positive association with LBP: too low seat height (OR = 1.44, 95% CI: 1.13–1.48; *P *< 0.01); too low seat backrest height (OR = 1.33, CI: 1.04–1.70; *P *< 0.05); seat backrest being too curved (OR = 1.74, 95% CI: 1.18–2.57; *P *< 0.05); seat backrest being too flat (OR = 1.30, 95% CI: 1.04–1.64; *P* < 0.05); too shallow seat depth (OR = 1.31, 95% CI: 1.01–1.70; *P *< 0.05); too low desk height (OR = 1.27, 95% CI: 1.01–1.60; *P *< 0.05); too far seat-to-black(board) distance (OR = 1.69, 95% CI: 1.32–2.18; *P *< 0.001); difficulty in viewing the (black)board (OR = 2.03, 95% CI:1.46–2.83; *P *< 0.001), too much homework (OR = 1.63, 95% CI: 1.28–2.07; *P *< 0.001); carrying a schoolbag for more than 60 min/day (OR = 1.77, 95% CI: 1.35–2.33; *P *< 0.001) and carrying a schoolbag on one shoulder (OR = 1.31, 95% CI: 1.05–1.64; *P *< 0.05).

The results of univariate logistic regression analyses of psychosocial factors associated with LBP are shown in Table 5. Schoolchildren reporting higher scores on “negative” behaviour subscales (i.e. hyperactivity, emotional symptoms, conduct problems and peer problems) were significantly more likely to experience LBP. Moreover, children reporting higher “total difficulties” score had an approximately threefold increased odds of reporting LBP. Prosocial behaviour was not associated with LBP.

### Multivariate analyses

The results from multivariate logistic regression analysis are presented in Table 6. Six factors including female gender (OR = 1.48, 95% CI:1.23–1.79;* P *< 0.01), family member with back pain (OR = 1.82, 95% CI: 1.40–2.36;* P *< 0.001), difficulty in viewing the (black)board (OR = 1.50, 95% CI: 1.13–1.99;* P *< 0.01), too much homework (OR = 1.47, 95% CI: 1.09–1.99;* P *< 0.05), carrying a schoolbag for more than 30 min/d (OR = 1.37, 95% CI: 1.01–1.85; *P *< 0.05), and high emotional symptoms (OR = 2.28, 95% CI: 1.54–3.39; *P *< 0.001) were independently associated with LBP.

## Discussion

This is one of the largest epidemiological studies so far conducted to evaluate LBP and its various contributing factors in school aged children. It extends the body of knowledge about the issue in this age group in developing countries. Our findings confirm that LBP is frequent in Iranian schoolchildren, which is in line previous reports.^3,4,14,16,20,21^ Individual factors including gender (being female) and family member with back pain were independently associated with the occurrence of LBP. School-related variables such as difficulty in viewing the (black)board, too much homework and carrying a schoolbag for more than 30 min/d, as well as psychosocial factors (high emotional symptoms) were also independently associated with the presence of LBP. Physical activity and leisure time physical activity (including having a job, time spent on sport activities, watching television, using a computer or playing games) and schoolbag weight were not associated with LBP.

Among demographic factors, gender was an independent factor predicting LBP in schoolchildren. Overall, girls had a 50% increased odds of reporting LBP compared with boys. This is similar to several previous reports, which have shown a higher prevalence of LBP in girls than in boys.^3,14,15,20^ The higher prevalence rate of symptoms in girls may be attributed to earlier female puberty and its associated hormonal changes^22^; while on the other hand, boys may have a tendency to under report or worry less about this problem.^6^ It therefore appears that girls are at greater risk of developing LBP as compared with boys. This highlights the need for specifically designed interventional programs for reducing the prevalence of LBP in this group. Moreover, a positive association was found between symptom reporting of family members and LBP among schoolchildren in this study, which is also similar to previous reports.^20^ In contrast, other personal factors including age and BMI as well as physical activity and leisure time physical activity variables (such as having a job and time spent on sport activity, using a computer and playing games) were not found to contribute to any increased or decreased risk for LBP in this study. These findings are in line with some recent reports from developed countries,^4,14^ which have shown no significant association between physical leisure activity variables and LBP.

As with the schoolbag carriage variables examined in this study, time spent carrying a schoolbag each day was the only factor that was independently positively associated with LBP. This finding is similar to several previous reports,^11,12^ which have found a positive relationship between time spent carrying schoolbags and the occurrence of LBP. Also, it should be noted that although the schoolbag weight has been described by some authors as one of the possible factors affecting musculoskeletal pain in schoolchildren,^11^ this was not the case in the present study. The lack of association between LBP and schoolbag weight (which was expressed as a percentage of body weight) in our study is consistent with several recent work.^4,6,9,23,24^ Given the contradictory findings in the literature, these findings provide additional evidence that the schoolbag weight may not be the only factor associated with back pain amongst schoolchildren and other schoolbag carriage variables such as the time spent carrying schoolbags to and from school each day by schoolchildren should also be taken into account.

It is also of interest to note that inappropriate classroom furniture and lay out design were found to be possible contributing factors to LBP in our study. Difficulty in viewing the (black)board was found to be independently associated LBP. Moreover, several features of classroom furniture including seat height (too low), seat backrest height (too low), seat backrest curvature (too curved/flat), seat depth (too shallow), and desk height (too low) had significant associations with the occurrence of LBP in univariate analyses, but not as independent factors in multivariate analysis. Similar to these findings, Murphy et al^4^ found a positive association between LBP and chair height being too low; while Trevelyan and Legg^9^ reported that LBP was significantly associated with the backrest of the chair being too curved and to the desk height being too low. The results of a recent study among Iranian schoolchildren also showed a considerable mismatch between anthropometric dimensions of children and the existing classroom furniture.^25^ Taken together, these findings highlight the fact that the classroom furniture and lay out design must be based on the anthropometric needs of schoolchildren to reduce the occurrence of musculoskeletal pain in this group.

With regard to the psychosocial factors, the results of the present study showed that the presence of emotional symptoms (calculated using the SDQ) was independently positively associated with the prevalence of LBP in Iranian schoolchildren. Similar findings have been reported by Watson et al^3^ and Murphy et al^4^ among 11–14 years old schoolchildren in the United Kingdom and by Trevelyan and Legg^9^ among the schoolchildren with the same age in New Zealand. Other authors have also reported significant relationships between psychosocial factors and the occurrence of back pain in schoolchildren.^2,14^ Based on these findings, it seems that psychological or psychosocial factors should be given a more prominent role when it comes to evaluating musculoskeletal pain in schoolchildren.

The present study has an advantage that one investigator was present during data collection in order to ensure all schoolchildren were able to understand and answer the questionnaires, and therefore to decrease the likelihood of bias from non-participation. This also prevented observer error as opposed to studies in which there was separate observer for each case (i.e. self-reported or parental-assisted reporting). However, the current findings should be considered in the context of the cross-sectional data collection, and whilst a large dataset was possible, causality cannot be ascertained. It should also be highlighted that the data rely on the accuracy and reliability of self-report. However, as pointed out by several authors, this may be the only measure to understand whether and how the schoolchildren feel any pain or discomfort.^26^ There are also limitations regarding the generalizability of the findings as the data presented here in this study are from one city of Iran, and thus further studies in other parts of the country and the world are recommended.

## Conclusion

In conclusion, this study extends the body of knowledge on LBP to include schoolchildren in developing countries. The main finding of the study was that LBP is frequent in Iranian schoolchildren, which is in line with reports from developed countries. Girls and those who had a family member with back pain were more likely to report LBP. Both physical (i.e. school-related items) and psychosocial variables (i.e. emotional symptoms) influenced the risk for LBP among schoolchildren, which emphasises the need for ergonomic and behaviour-based interventions in order to reduce such complaints in this group. The association of LBP with emotional symptoms was stronger than the association of LBP with physical factors, which highlights the importance of psychosocial interventions in reducing LBP in schoolchildren.

## Ethical approval

The study protocol was reviewed and approved by the local ethics review committee of the Tabriz University of Medical Sciences, Iran.

## Competing interests

There are no conflicts of interest.

## Authors’ contributions

ID involved in the conceptualization and designing of the study. AA involved in the data collection. MA involved in the data analysis and drafting the manuscript.

## Acknowledgments

This project was funded by the Tabriz University of Medical Sciences (Grant number: 5/53/511).

**Table 1:** Physical leisure activities, use of schoolbag and SDQ scores among the study population

**Variables**	**No. (%)**	**Mean (SD)**	**Range**
**Physical leisure activities**			
Plying sport (h/wk)	1294 (80.3)	4.8 (6.1)	0.3–48
Using a computer (h/wk)	1059 (65.7)	7.1 (8.5)	0.3–49
Playing games (h/wk)	887 (55.0)	6.2 (8.1)	0.15–63
Watching TV (h/wk)	1381 (85.7)	12.9 (12.4)	0.2–50
Having Job (h/wk)	48 (3.0)	2.4 (2.8)	2–20
**Schoolbag carriage variables**			
Schoolbag weight (kg)	–	3.1 (1.6)	0.26–11.5
Schoolbag as % body weight	–	6.5 (3.6)	1.44–24.13
Time spent carrying schoolbag (min)	–	49.8 (40.2)	5–120
Type of schoolbag			
*Backpack*	1244 (77.2)	–	–
*Brief case/satchel*	367 (22.8)	–	–
Method of schoolbag carriage			
*On both shoulders*	674 (41.8)	–	–
*On one shoulder*	762 (47.3)	–	–
*By hands*	175 (10.9)	–	–
Method of travel to/from school			
*Walk*	406 (25.2)	–	–
*Bus/car*	1159 (71.9)	–	–
*Bike*	46 (2.9)	–	–
**SDQ scale**			
Total difficulties			
*Normal*	868 (53.8)	–	–
*Borderline*	309 (19.2)	–	–
*Abnormal*	436 (27.0)	–	–
Emotional symptoms			
*Normal*	914 (56.7)	–	–
*Borderline*	231 (14.3)	–	–
*Abnormal*	468 (29.0)	–	–
Conduct problems			
*Normal*	914 (56.7)	–	–
*Borderline*	283 (17.5)	–	–
*Abnormal*	416 (25.8)	–	–
Hyperactivity			
*Normal*	1210 (75.0)	–	–
*Borderline*	210 (13.0)	–	–
*Abnormal*	193 (12.0)	–	–
Peer problems			
*Normal*	538 (33.3)	–	–
*Borderline*	366 (22.7)	–	–
*Abnormal*	709 (44.0)	–	–
Prosocial behaviour			
*Normal*	1212 (75.1)	–	–
*Borderline*	185 (11.5)	–	–
*Abnormal*	216 (13.4)	–	–

**Table 2:** Characteristics of the LBP among the study population

**Variable**	**LBP**	
	**Boys**	**Girls**
Prevalence of LBP, No. (%)	218 (29.0)	335 (39.0)
Pain severity (scale 0–5), mean (SD)	2.5 (1.25)	3.1 (1.32) ^a^
Disruption of normal activities, No. (%)	115 (52.9)	192 (57.1)
Absent from school, No. (%)	38 (17.5)	32 (9.5)
Visit the doctor, No. (%)	23 (10.6)	48 (14.3)

Abbreviation: LBP, low back pain. ^a^ Ordinal logistic regression (*P* < 0.05).

**Table 3:** Demographic and physical leisure activity variables and risk of LBP using univariate logistic regression

	**% With LBP**	**OR**	**95% CI**	***P***
Age (y)				
≤13	35	1.0	(Referent)	
>13	34	1.05	(0.86–1.30)	0.621
Gender				
Boy	29	1.0	(Referent)	
Girl	39	1.57	(1.28–1.94)	0.001
BMI (kg/m^2^)				
<17.33	31	1.0	(Referent)	
17.33–19.38	34	1.15	(0.84–1.56)	0.362
19.39–22.04	36	1.25	(0.92–1.70)	0.140
>22.04	34	1.16	(0.85–1.58)	0.322
Family member with back pain				
No	33	1.0	(Referent)	
Yes	40	1.83	(1.48–2.27)	0.001
Playing sport (h/wk)				
<1	37	1.0	(Referent)	
1–3	33	0.83	(0.65–1.07)	0.150
>3	33	0.84	(0.65–1.08)	0.170
Using a computer (h/wk)				
<1	34	1.0	(Referent)	
1–4	35	1.05	(0.83–1.35)	0.680
>4	34	1.03	(0.80–1.34)	0.820
Playing games (h/wk)				
<1	34	1.0	(Referent)	
1–2	38	1.2	(0.92–1.55)	0.180
>2	33	0.96	(0.75–1.22)	0.710
Watching TV (h/wk)				
<3	33	1.0	(Referent)	
3–12	33	1.02	(0.79–1.31)	0.900
>12	37	1.17	(0.91–1.50)	0.216
Part time job				
No	34	1.0	(Referent)	
Yes	35	1.05	(0.57–1.91)	0.880

Abbreviations: LBP, low back pain; BMI, body mass index; OR, odds ratio.

**Table 4:** School-related variables and risk of LBP using univariate logistic regression

	**% With LBP**	**OR**	**95% CI**	***P***
**Classroom furniture and layout design**				
Seat height				
Just right	32	1.0	(Referent)	
Too high	47	1.86	(0.92–3.76)	0.084
Too low	41	1.44	(1.13–1.84)	0.004
Seat backrest height				
Just right	33	1.0	(Referent)	
Too high	35	1.12	(0.62–2.02)	0.700
Too low	39	1.33	(1.04–1.70)	0.020
Seat backrest inclination				
Just right	32	1.0	(Referent)	
Too high	40	1.20	(0.84–1.72)	0.320
Too low	44	1.24	(0.82–1.72)	0.200
Seat backrest curvature				
Just right	31	1.0	(Referent)	
Too curved	44	1.74	(1.18–2.57)	0.010
Too flat	37	1.30	(1.04–1.64)	0.020
Seat depth				
Just right	33	1.0	(Referent)	
Too deep	35	1.08	(0.64–1.81)	0.078
Too shallow	39	1.31	(1.01–1.70)	0.040
Seat width				
Just right	33	1.0	(Referent)	
Too wide	43	1.60	(0.93–2.35)	0.069
Too narrow	38	1.26	(0.98–1.62)	0.075
Desk height				
Just right	32	1.0	(Referent)	
Too high	39	1.34	(0.85–2.10)	0.205
Too low	38	1.27	(1.01–1.60)	0.038
Seat pan inclination				
Just right	34	1.0	(Referent)	
Too backward	38	1.18	(0.82–1.72)	0.374
Too forward	37	1.17	(0.78–1.76)	0.454
Seat-to-(black)board distance				
Just right	31	1.0	(Referent)	
Too near	35	1.18	(0.79–1.74)	0.420
Too far	44	1.69	(1.32–2.18)	0.001
Viewing the (black)board				
Very easy	27	1.0	(Referent)	
Neutral	40	1.82	(1.54–2.27)	0.001
Very difficult	43	2.03	(1.46–2.83)	0.001
Homework				
Just right	30	1.0	(Referent)	
Not enough	40	1.35	(0.90–1.74)	0.186
Too much	41	1.63	(1.28–2.07)	0.001
**Schoolbag carriage variables**				
Type of schoolbag				
Backpack	34	1.0	(Referent)	
Briefcase	33	0.94	(0.72–1.25)	0.690
Shoulder bag	42	1.37	(0.85–2.22)	0.200
Schoolbag weight (as % body weight)				
≤10%	31	1.0	(Referent)	
>10%	33	1.14	(0.69–1.87)	0.610
Time spent carrying schoolbag (min/day)				
≤30	30	1.0	(Referent)	
30–60	35	1.27	(0.98–1.64)	0.071
>60	43	1.77	(1.35–2.33)	0.001
Method of schoolbag carriage				
Both shoulders	32	1.0	(Referent)	
One shoulder	38	1.31	(1.05–1.64)	0.020
By hands	30	0.92	(0.64–1.32)	0.640
Method of travel to/from school				
Bus/car/Bike	32	1.0	(Referent)	
Walk	35	0.87	(0.69–1.11)	0.269

Abbreviations: LBP, low back pain; OR, odds ratio.

**Table 5:** Psychosocial factors and risk of LBP using univariate logistic regression

**SDQ dimensions**	**% with LBP**	**OR**	**95% CI**	***P***
**Strengths**				
Prosocial behaviour				
Low	35	1.0	(Referent)	
Medium	31	0.86	(0.62–1.21)	0.390
High	36	1.04	(0.77–1.41)	0.790
**Difficulties**				
Emotional symptoms				
Low	26	1.0	(Referent)	
Medium	36	1.67	(1.23–2.26)	0.001
High	50	2.96	(2.35–3.75)	0.001
Conduct problems				
Low	29	1.0	(Referent)	
Medium	39	1.55	(1.18–2.05)	0.002
High	44	1.93	(1.52–2.46)	0.001
Hyperactivity				
Low	31	1.0	(Referent)	
Medium	39	1.43	(1.06–1.93)	0.021
High	50	2.25	(1.66–3.06)	0.001
Peer problems				
Low	31	1.0	(Referent)	
Medium	35	1.21	(0.91–1.60)	0.196
High	37	1.33	(1.05–1.69)	0.019
Total difficulties				
Low	26	1.0	(Referent)	
Medium	39	1.84	(1.40–2.43)	0.001
High	48	2.72	(2.14–3.47)	0.001

Abbreviations: LBP, low back pain; OR, odds ratio.

**Table 6:** Predictors of LBP among the study population using multivariate logistic regression

**Variables**	**OR**	**95% CI**	***P***
Gender			
Boy	1.0	(referent)	
Girl	1.48	(1.23–1.79)	0.002
Family member with back pain			
No	1.0	(referent)	
Yes	1.82	(1.40–2.36)	0.001
Viewing the (black)board			
Very easy	1.0	(referent)	
Neutral	1.22	(0.79–1.90)	0.367
Very difficult	1.50	(1.13–1.99)	0.005
Homework			
Just right	1.0	(referent)	
Not enough	1.32	(0.90–1.96)	0.159
Too much	1.47	(1.09–1.99)	0.013
Time spent carrying schoolbag (min/d)			
≤30	1.0	(referent)	
30–60	1.37	(1.01–1.85)	0.043
>60	1.52	(1.10–2.11)	0.011
Emotional symptoms			
Low	1.0	(referent)	
Medium	1.22	(0.81–1.84)	0.341
High	2.28	(1.54–3.39)	0.001

Abbreviations: LBP, low back pain; OR, odds ratio.
